# Total calcium absorption is similar from infant formulas with and without prebiotics and exceeds that in human milk-fed infants

**DOI:** 10.1186/1471-2431-12-118

**Published:** 2012-08-07

**Authors:** Penni D Hicks, Keli M Hawthorne, Carol L Berseth, John D Marunycz, James E Heubi, Steven A Abrams

**Affiliations:** 1 Rice University, Houston, TX, USA; 2 Department of Agriculture/Agricultural Research Service Children's Nutrition Research Center, Department of Pediatrics, Baylor College of Medicine, Houston, TX, USA; 3 Mead Johnson Nutrition, Evansville, IN, USA; 4 Cincinnati Children's Hospital Medical Center, Cincinnati, OH, USA

## Abstract

**Background:**

1) To evaluate calcium absorption in infants fed a formula containing prebiotics (PF) and one without prebiotics (CF). 2) To compare calcium absorption from these formulas with a group of human milk-fed (HM) infants.

**Methods:**

A dual tracer stable isotope method was used to assess calcium absorption in infants exclusively fed CF (n = 30), PF (n = 25) or HM (n = 19). Analysis of variance was used to analyze calcium intake, fractional calcium absorption, and the amount of calcium absorbed.

**Results:**

Calcium intake (Mean ± SEM) for PF was 534 ± 17 mg/d and 557 ± 16 mg/d for CF (p = 0.33). Fractional calcium absorption was 56.8 ± 2.6 % for PF and 59.2 ± 2.3 % for CF (p = 0.49). Total calcium absorbed for PF was 300 ± 14 mg/d and 328 ± 13 mg/d for CF (p = 0.16). For HM infants calcium intake was 246 ± 20 mg/d, fractional calcium absorption was 76.0 ± 2.9 % and total calcium absorbed was 187 ± 16 mg/d (p <0.001, compared to either PF or CF).

**Conclusions:**

Despite lower fractional calcium absorption of CF and PF compared to HM, higher calcium content in both led to higher total calcium absorption compared to HM infants. No significant effect of prebiotics was observed on calcium absorption or other markers of bone mineral metabolism.

## Background

The standard for nutrient intake and bioavailability in the first months of life is the exclusively human milk-fed infant. Because of the possibility of lower bioavailability, the quantity of minerals important for bone development, including calcium, in infant formulas are greater than those found in human milk (HM). Concentration ranges for calcium in infant formulas are set by statute in the United States and many countries. Because of these higher nutrient concentrations, it is not possible to directly compare the intrinsic bioavailability of calcium from infant formulas to that of HM. Nonetheless, it is important to assure that the total amount of calcium absorbed from any infant formula is at least equal to that provided by HM and to evaluate calcium absorption as changes are made to infant formula composition.

Prebiotics are currently routinely added to infant formulas. A prebiotic is a non-digestible food ingredient that brings about specific changes in the composition and/or activity of the gastrointestinal microbiota that confer benefits upon host well-being and health [1]. Prebiotics potentially have several beneficial effects on neonatal intestinal development, including promoting the establishment of beneficial microbiota, protecting against infection, promoting intestinal adaptation to the extrauterine environment and compensating for the developmental immaturity of the intestine [2].

Prebiotics enhance calcium absorption in adolescents [3,4]. This may relate to lower proximal colon pH from short chain fatty acids produced by prebiotics which might increase the amount of calcium that is present in the soluble phase available for absorption [5]. Alternatively, prebiotics may have an overall trophic effect on the intestinal mucosa leading to an increase in calcium absorption [5,6].

No studies exist of the effects of prebiotic administration on calcium absorption in infants. The objective of the current study was to evaluate calcium absorption in infants fed formula with or without prebiotics and to compare this absorption with that of infants fed HM, a natural source of prebiotics.

## Methods

### Study design and population

We conducted a multi-center, double-blind randomized controlled trial to assess calcium absorption in healthy infants fed a formula with prebiotics (Prebiotic Formula, PF) or without prebiotics (Control Formula, CF). At the time of the study, 2003-2008, prebiotics were not included in most cow milk-based routine formulas marketed in the United States. Infants were recruited from five hospitals in the United States via open public advertisements. Subjects included in the study were term infants (37-42 weeks gestational age) with a birth weight ≥ 2500 g, who at the time of enrollment were consuming a cow milk-based, lactose-containing formula. Subjects were recruited and enrolled in the study prior to 10 weeks of age. Exclusion criteria included a history of underlying disease or congenital malformation that was likely to interfere with the normal growth and development or the evaluation of calcium absorption, evidence of formula intolerance or poor intake, and consuming juices and/or solid foods (including cereals and baby food).

A non-randomized HM-fed group from one of the study sites (Baylor College of Medicine) was used for comparison. The inclusion and exclusion criteria were similar to those of the formula-fed groups with the exception of requiring exclusive consumption of maternal HM.

The study was approved by the Institutional Review Boards for Human Subject Research of Baylor College of Medicine and Affiliated Hospitals (Houston, TX), Cincinnati Children’s Hospital Medical Center (Cincinnati, OH), Western (Coralville, Iowa), and University of Louisville and Boys Town National Research Hospital (Omaha, NE). Informed written consent was obtained from the parents of all infants prior to study initiation.

### Study diet

Formula-fed infants were fed either a cow milk-based non-prebiotic containing control formula (CF), (marketed at the time of study as Enfamil LIPIL®, Mead Johnson Nutrition, Evansville, IN), or the same formula with added prebiotics. This prebiotic-containing formula (PF) contained galactooligosaccharides (GOS) and polydextrose (PDX) in a 1:1 at 4 g/L. Human milk-fed infants consumed HM along with daily multiple vitamin drops providing vitamins A and C as well as 400 IU of vitamin D.

Subjects were enrolled and randomized to study formula between 56-70 days of age. After randomization assignment, participants consumed either CF or PF as their primary source of nutrition for a minimum of 14 days. The participants were provided with 2-3 cases of ready-to-feed formula; the amount of feeding was left at the discretion of the parent/caregiver. Human milk-fed infants who had consumed HM from birth also were enrolled between 56-70 days of age.

### Calcium absorption study

**Figure 1:**
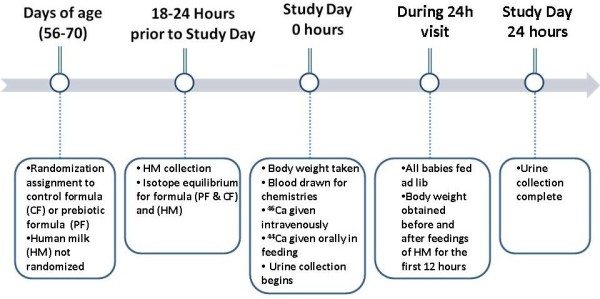
Timeline of Infant Evaluation.

After two weeks of study formula consumption (Figure 1), subjects were admitted for a 24-hr inpatient hospital stay where calcium absorption was measured by a dual tracer stable isotope method [7-10]. In this technique, accepted as the standard for calcium absorption measurements, one low-abundance calcium stable isotope, ^44^Ca, was given orally in the formula or human milk, and another isotope, ^46^Ca, was given intravenously.

Between 18 and 24 hours prior to ingestion, 60 mL of CF, PF or HM was mixed with ^44^Ca (3 mg) and refrigerated until the study began to allow for equilibration. Upon admission, the subject was fitted with a urine collection bag (First-Time Urine Specimen Collector, Hollister Incorporated, Libertyville, IL). Diapers applied over the urine bag were weighed prior to and after use to determine the amount of any urine spilled. The infants were then given ^46^Ca as the chloride salt (0.01 mg) intravenously over 1 minute via a standard infusion technique.

The feeding containing the premixed oral isotope was fed to the infant via feeding bottle; immediately following consumption of this, an additional 60 mL of unlabeled feeding or maternal HM via bottle was given. Urine collection began at the time of intravenous isotope administration and was collected for 24 hours following the isotope administration.

Fractional calcium absorption was calculated as the ratio of the oral to the intravenous tracer recovered in the urine during the 24-hr collection multiplied by 100 to give a percentage absorbed. Based on common usage, the term fractional absorption refers to the percent dose that was absorbed, although this is not a true “fraction” as mathematically defined. Others use the term calcium absorption efficiency to describe the percent absorption from a dose or meal. Total calcium absorbed was calculated by multiplying the calcium intake by the fractional absorption.

Calcium intake for formula-fed infants was determined based on the measured intake during the study and the labeled calcium concentration. For human milk-fed infants, a concentration of 30 mg/100 mL was used based on individual samples analyzed from mothers in this study. This was multiplied by the milk volume intake determined by test weighing for each individual infant.

Isotopic enrichment was determined at Baylor College of Medicine using a magnetic sector Finnigan MAT 261 thermal ionization mass spectrometer (Bremen, Germany) [4,7,8].

### Anthropometrics and chemical analysis of serum samples

Subjects were weighed at enrollment and at the start of the 24-hr inpatient stay. During the inpatient stay, 1 mL of blood was obtained for chemical analysis. Serum calcium, phosphorus, and alkaline phosphatase activity were analyzed at a central clinical laboratory at Cincinnati Children’s Hospital. Serum 25-OH vitamin D levels were measured by radioimmunoassay using the DiaSorin (Stillwater, MN) Assay.

### Statistics

Total calcium absorption was the primary study outcome value. The sample size was determined so that the study would have a power of 80 % to detect a clinically relevant difference in calcium absorption of 10 mg/100 kcal between the two formula study feeding groups when testing at an alpha level of 0.05. Assuming a standard deviation of 11.4 mg/100 kcal, 22 infants per group were needed.

All statistical tests were conducted at an alpha level of 0.05. Analysis of variance was used to analyze calcium intake, fractional calcium absorption, and the amount of calcium absorbed. Feeding group was the only term included in the statistical model. If the overall F test was rejected, pair-wise comparisons of the feeding groups were made. Fisher’s exact test was used to compare the distribution of gender among the feeding groups. Analysis of variance was used to analyze age and weight at the time of calcium absorption assessment. Feeding group was the only term included in the model for the age analysis, while feeding group and gender were the terms included in the model for the weight analysis.

Analysis of variance also was used to analyze serum nutrient concentrations and alkaline phosphatase activity. Feeding group was the only term in the model and the HM group was not included in the analysis. A post hoc exploratory analysis was performed on formula-fed infants to evaluate the relationship between calcium absorption and 25-OHD level. The calcium absorption results were compared between infants with 25-OHD levels ≤ 20 ng/mL and those with levels > 20 ng/mL using analysis of variance. A cut-off of normal of 20 ng/mL for 25-OHD was used based on the recent Institute of Medicine report [11]. Data are presented as Mean ± SEM.

## Results

Of 72 formula-fed infants enrolled in the study, 55 completed the calcium absorption evaluation. The number of infants who completed the study was similar across of each of the formula groups. Subject characteristics of the 74 infants, which includes the 19 HM-fed infants who were evaluated are shown in Table 1. Twenty-five infants consumed PF, 30 infants consumed CF. There were no significant differences among feeding groups in age or weight at the time of calcium absorption assessment.

**Table 1:** Subject Characteristics at Time of Calcium Absorption Study

	**Prebiotic Formula N = 25**	**Control Formula N = 30**	**Human Milk Reference N = 19**
Age, day of life	78 ± 1	77 ± 1	78 ± 1
Weight, g	5829 ± 157	5811 ± 140	5809 ± 176
Gender, Male/Female	17/8	16/14	11/8

Includes only infants with a calcium absorption result. No statistical differences detected among the feeding groups. Data are Mean ± SEM.

Calcium intake, fractional calcium absorption and total calcium absorption were similar between PF and CF groups, as shown in Table 2. Calcium absorbed per 100 kcal was 44.3 ± 1.8 mg for PF group and 46.2 ± 1.6 mg for the CF group.

**Table 2:** Calcium absorption in study subjects

	**Prebiotic Formula N = 25**	**Control Formula N = 30**	**Human Milk Reference N = 19**
Calcium intake, mg/d	534 ± 17	557 ± 16	246 ± 20*
Percent calcium absorption	56.8 ± 2.6	59.2 ± 2.3	76.0 ± 2.9*
Total calcium absorbed^a^, mg/d	300 ± 14	328 ± 13	187 ± 16*

Data are Mean ± SEM.

* Significantly different from both PF and CF groups (p < 0.001).

^a^Total calcium absorbed calculated as (calcium intake [mg/day]) x (fractional absorption).

Calcium intake, fractional calcium absorption and total calcium absorption from the HM group were significantly different from both PF and CF groups. Calcium intake for the HM group was 246 ± 20 mg/d (p <0.001, compared to PF and CF groups), fractional calcium absorption was 76.0 ± 2.9 % (p < 0.001, compared to PF and CF groups) and total calcium absorbed was 187 ± 16 mg/d (p < 0.001, compared to PF and CF groups). Calcium absorbed per 100 kcal was 33.7 ± 2.0 mg for HM group.

Serum nutrient concentrations and alkaline phosphatase activity of infants with calcium absorption results are shown in Table 3. Serum calcium was significantly higher in the PF group than the CF group. There were no significant differences in serum phosphorus or alkaline phosphatase activity between the PF and CF groups. No statistical comparisons of the laboratory values were made between the HM group and the PF or CF groups.

**Table 3:** Laboratory Values

	**Prebiotic Formula N = 20**	**Control Formula N = 29**	**Human Milk Reference**^**a**^N = 16**
Serum calcium, mg/dL	10.3 ± 0.1	9.9 ± .01***	10.2 ± 0.2
Serum phosphorus, mg/dL	6.9 ± 0.1	6.7 ± 0.1	5.9 ± 0.2
Serum alkaline phosphatase activity, IU/L	326 ± 19	331 ± 16	282 ± 21
Serum sodium, mEq/L	140.2 ± 0.6	139.6 ± 0.5	-
Serum potassium, mEq/L	5.5 ± 0.1	5.4 ± 0.1	-
Serum creatinine, mEq/L	0.29 ± 0.03	0.30 ± 0.02	-
Serum magnesium, mg/dL	2.03 ± 0.03	2.00 ± 0.03	-
Serum 25-hydroxyvitamin D, ng/mL	30.5 ±1.8	28.7 ±1.5	28.7 ± 2.8

Data are Mean ± SEM.

Includes only infants with a calcium absorption result.

^a^No statistical comparisons to formulas were made.

**N = 18 for serum 25-hydroxyvitamin D.

*** p = 0.01, PF versus CF.

Serum 25-hydroxyvitamin D (25-OHD) was 30.5 and 28.7 ng/mL in the PF and CF formula-fed infants (Table 3) and was not significantly different between groups (p = 0.42). The relationship between both fractional and total calcium absorptions and 25-OHD level was evaluated. For formula-fed infants, 49 infants had both calcium absorption and 25-OHD results. Of these 49 infants we found the 7 infants with a 25-OHD value ≤ 20 ng/mL had a significantly lower total calcium absorption than the 42 infants with a 25-OHD value > 20 ng/mL (247 ± 27 mg/d vs 327 ± 11 mg/d, p = 0.01). Fractional calcium absorption was also lower (50.3 ± 4.3 % vs 59.9 ± 1.7 %, p = 0.04).

This difference was not seen for the 18 human milk-fed infants with both calcium absorption and 25-OHD results. Both total (185.5 ± 24.3 mg/d vs 188.5 ± 15.1 mg/d, p = 0.91) and fractional calcium absorption (80.4 ± 6.7 % vs 75.5 ± 4.2 %, p = 0.54) were virtually identical for the 5 infants with 25-OHD values ≤ 20 ng/mL and the 13 infants with 25-OHD values > 20 ng/mL (the first values shown are for the infants with the lower 25-OHD values).

## Discussion

We found a higher net calcium absorption from infant formulas with or without prebiotics. The presence of prebiotics in the formula did not significantly affect calcium bioavailability. Calcium absorption fractions (efficiency) for PF and CF in the current study was similar to those seen with previous dual tracer isotope evaluations of infant formulas [12,13].

The absorption of calcium from HM in this study was higher than the value of 61 ± 23 % reported previously for older (4-7 month old), mixed-fed infants [12]. The higher values for calcium absorption in this study may be related to the effect of solid foods in older infants. These solid foods may lower absorption in the previously studied older infants compared to those studied in this report. The higher calcium intake in the older infants previously reported may also be responsible for the lower absorption values in that study because as calcium intake increases absorption efficiency decreases [12]. These data are also consistent with a calcium absorption efficiency of 60 % published by Nelson and Foman [14]. Comparison of the data with the mass balance data however is not precise due to the differing methodologies [14].

Regardless of the presence of prebiotics, total calcium absorption from a cow milk-based formula exceeded that of HM-fed infants. Since the concentration of calcium in all standard infant formulas markedly exceeds that of HM by regulatory statute in the United States, it is impossible to directly compare the relative bioavailability of calcium in human milk and infant formulas [15].

No effect of prebiotics on calcium absorption was found in this study, demonstrating that the calcium status in infants fed a prebiotic-supplemented formula was similar to that of infants fed a marketed control formula. Prebiotics have been found to increase calcium absorption in adolescents consuming a mixture of commercially available oligofructose-enriched inulin [4]. The finding of no effect of prebiotics on calcium absorption in our study may be related to the formulation of the PF, containing a mixture of prebiotics (GOS and PDX) rather than a single prebiotic product (inulin). Differences in gastrointestinal pH between infants and adolescents also could have an effect on the impact of prebiotics on mineral absorption.

None of the serum nutrient concentrations or alkaline phosphatase activity laboratory values were outside of the range of normal. While serum calcium was significantly higher in the PF group compared with the CF group, the difference was not clinically significant. Vitamin D status was adequate as evidenced by the mean 25-OHD values, although it is important to note that a relationship between serum 25-OHD levels and bone health outcomes in the first months of life is not well established [11].

There were a few infants in both the formula group (7 of 49) and HM group (5 of 18) with 25-OHD values at or below 20 ng/mL. Of note is that for the formula group, but not the human milk-fed group, the fractional and total calcium absorptions were significantly lower for these infants. These fractional and total calcium absorption results are consistent with a targeted 25-OHD level of about 20 ng/mL or greater, as recommended for older children and adults by the Institute of Medicine [11]. However, caution is advised in interpreting these results. Too few infants had 25-OHD values at or below 20 ng/mL to convincingly demonstrate a meaningful effect and this study was not designed to evaluate this issue. Nonetheless, our results suggest that further evaluation of the effects of low 25-OHD levels on calcium absorption in infants should be considered.

Although we used an extrinsic tracer, this method is fully validated for milk sources and it is unlikely that any significant proportion of the HM or formula did not equilibrate with the tracer as this approach has been widely used and validated for milk-based products [16].

In conclusion, despite lower fractional calcium absorption from CF and PF compared with HM, the higher calcium content in both led to higher 24-hour calcium absorption compared with that of HM infants. Whether the higher net calcium absorption from the formulas is beneficial is unknown. No effect of prebiotics on calcium absorption was seen with the PF, demonstrating that the addition of prebiotics supports calcium status in infants fed a prebiotic-supplemented formula similar to that of a marketed control formula.

## Competing interests

Funding for this study was provided by Mead-Johnson Nutrition. Drs. Hicks, Heubi and Abrams and Ms. Hawthorne have no competing interests. Mr. Marunycz and Dr. Berseth are employees of Mead-Johnson Nutrition.

## Authors’ contributions

All authors except JDM assisted in the design of the study, performed the data collection, and prepared the manuscript. JEH, PDH, and KMH participated in data collection. JDM participated in the data analysis and manuscript preparation. All authors read and approved the manuscript prior to submission.

## Pre-publication history

The pre-publication history for this paper can be accessed here:

http://www.biomedcentral.com/1471-2431/12/118/prepub
